# Isolation of non-tuberculous mycobacteria among tuberculosis patients, a study from a tertiary care hospital in Lahore, Pakistan

**DOI:** 10.1186/s12879-021-06086-8

**Published:** 2021-04-24

**Authors:** Asifa Karamat, Atiqa Ambreen, Aamira Ishtiaq, Sabira Tahseen, Muhammad Aqeelur Rahman, Tehmina Mustafa

**Affiliations:** 1 Department of Tuberculosis and Chest Medicine, Gulab Devi Hospital, Lahore, Pakistan; 2 Department of Microbiology, Gulab Devi Hospital, Lahore, Pakistan; 3 National Tuberculosis Control Programme and National Tuberculosis Reference Laboratory, Islamabad, Pakistan; 4 Centre for International Health, Department of Global Public Health and Primary Care, University of Bergen, P.O. box 7804, N-5020 Bergen, Norway; 5 Department of Thoracic medicine, Haukeland University Hospital, Bergen, Norway

**Keywords:** Non-tuberculous mycobacteria, NTM pulmonary disease, NTM treatment, Mycobacterial species, Pulmonary tuberculosis, Acid-fast microscopy

## Abstract

**Background:**

There is scarce knowledge on the prevalence of diseases caused by non-tuberculous mycobacteria (NTM) in Pakistan. In the absence of culture and identification, acid-fast bacilli (AFB) causing NTM disease are liable to be misinterpreted as tuberculosis (TB). Introduction of nucleic acid amplification testing for *Mycobacterium tuberculosis* complex (MTBC) offers improved diagnostic accuracy, compared with smear microscopy, and also assists in differentiating MTBC from other mycobacteria. This study aimed to investigate the prevalence of NTM among patients investigated for TB and describe NTM disease and treatment outcomes at a tertiary care hospital in Pakistan.

**Methods:**

This is a retrospective study, data on NTM isolates among culture-positive clinical samples over 4 years (2016–19) was retrieved from laboratory records. Information on clinical specimens processed, AFB smear results, and for the AFB positive isolates, results of species identification for MTBC, and for NTM isolates, results of species characterization and drug susceptibility testing was collected. Additional clinical data including patient characteristics, treatment regimens, and outcomes were collected for patients with NTM disease treated at Gulab Devi Hospital, Lahore.

**Results:**

During the study period, 12,561 clinical specimens were processed for mycobacterial culture and 3673 (29%) were reported positive for AFB. Among these 3482 (95%) were identified as MTBC and 191 (5%) as NTM. Among NTM, 169 (88%) were isolated from pulmonary and 22 (12%) from extrapulmonary specimens. Results of NTM speciation were available for 60 isolates and included 55% (*n* = 33) *M. avium* complex and 25% (*n* = 15) *M. abscesses.* Among these patients, complete clinical records were retrieved for 12 patients with pulmonary disease including nine infected with *M. avium* complex and three with *M. abscessus*. All 12 patients had a history of poor response to standard first-line anti-TB treatment. Ten patients were cured after 18 months of treatment, whereas, one with *M. abscessus* infection died and another was lost to follow up.

**Conclusion:**

In TB endemic areas, NTM can be misdiagnosed as pulmonary TB leading to repeated failed anti-TB treatment and increased morbidity, emphasizing the need for improved diagnosis.

## Background

Non-tuberculous mycobacteria (NTM) are ubiquitous environmental acid-fast bacilli (AFB). Although NTM are not obligate pathogens, they share with *M. tuberculosis* complex (MBTC) the features of hardiness, hydrophobicity, aerosolization, and intracellular pathogenicity. Even though the lung is the most common organ involved, NTM can cause disease in other organs of the body [1]. The commonest extrapulmonary organs involved are lymph nodes, skin/soft tissue, and disseminated disease [2]. The probability of NTM disease increases with the extent of environmental exposure, immunosuppression, and pulmonary NTM disease often occurs with co-existent chronic lung diseases [3–5]. However, pulmonary NTM disease can also be seen in previously well individuals [6]. It is usually difficult to distinguish tuberculosis (TB) from NTM lung disease based on clinical and radiological features [6]. The pulmonary disease caused by NTM, is likely to be misinterpreted as pulmonary TB based on AFB microscopy in the absence of culture and species identification [7]. There are increasing reports of NTM worldwide [8–13] and the likelihood that a substantial number of sputum smear-positive cases might be due to NTM. In high TB endemic countries, the chances of missing NTM species are higher because of the higher pre-test probability of TB, scarce resources, limited laboratory capacity, and overburdened health systems. The information regarding their true incidence and prevalence in these countries is scarce [14]. This study aimed to investigate the prevalence of NTM and different NTM strains among the patients investigated for TB and to describe the disease characteristics and treatment outcome of patients with NTM at a tertiary care hospital in Pakistan.

## Methods

### Study setting

Pakistan is a high TB burden country. For diagnosis of TB, AFB microscopy services are available at more than 1500 health facilities across the country. Automated nucleic acid amplification (Xpert MTB/RIF) assay was introduced in 2011 at few health facilities in Pakistan. The testing facilities were gradually scaled up and were made available at more than 300 health facilities by 2019. Culture and drug sensitivity testing services are limited to only a few centers in the country. The study was conducted at Gulab Devi Hospital. It is a private not-for-profit large tertiary care hospital located in Lahore, the capital city of the country’s largest province. Gulab Devi hospital is a specialized TB Hospital and is equipped with Xpert MTB/RIF assay and TB culture facilities. Diagnosis of pulmonary TB is routinely made by sputum smear microscopy, and culture is not performed on all samples [15]. Culture is usually requested by the physicians when patients are not responding to standard anti-TB treatment to see if the patient is infected with drug-resistant strains. For extrapulmonary TB and other difficult-to-diagnose cases, culture may be requested for diagnosis.

Laboratory facilities for NTM speciation were established at the national TB reference laboratory Islamabad in 2017 and are offered free of cost for patients seeking care from the linked facilities. Facilities for drug susceptibility testing for NTM, are only offered by one reference laboratory in the private sector, and only selected patients are referred there to get a susceptibility pattern.

### Study design and data collection

This is a retrospective study. Routinely collected data was used for the study. All clinical isolates which were reported culture positive for AFB between 2016 and 2019 were included in the study. Culture results, Species identification report, drug susceptibility testing reports, and follow-up data were obtained from laboratory registers and patient files.

### Laboratory methods

Specimens were processed with Petroff’s method using the NALC-NaOH method at a final concentration of 4% NaOH [16]. Extrapulmonary samples from sterile sites were concentrated without decontamination. Processed sediment was inoculated on two slopes of Lowenstein-Jensen medium and a BACTEC Mycobacteria Growth Indicator Tube 960 (MGIT960; Becton Dickinson, Sparks, MD, USA). Smears of the processed sediment were stained with auramine and examined using a light-emitting diode fluorescence microscope [16].

All culture isolates were first examined for AFB on smear. All AFB positive isolates were tested for detection of MPT64 antigen, specific for MBTC, using the TB Ag MPT64 rapid test kit (SD Bioline, Kyonggi, Korea) [16]. All culture isolates which were AFB positive but negative for MBTC were shipped to the national TB reference laboratory Islamabad for NTM identification. At the national TB reference laboratory, freshly grown subcultures were tested again for MTBC. Culture isolates negative for MTBC were then processed first for primary identification and characterization of species for the most common mycobacterial species using reverse hybridization-based line probe assay, GenoType Mycobacterium CM (Hain Lifescience, Nehren Germany).

Drug susceptibility testing was done at Aga Khan University Hospital, Karachi. Broth microdilution was used to perform drug susceptibilities using 96 well sensitive plates (TREK Diagnostic Systems Ltd., UK) as per the manufacturer’s recommendations. Susceptibility data thus obtained was interpreted according to the Clinical and Laboratory Standards Institute’s criteria [17].

### Statistical analysis

The data were entered into SPSS version 20 and cleaned for further analysis. A chi-square test was performed to show the differences between the pulmonary and extrapulmonary patient groups. A *p*-value of less than 0.05 was considered statistically significant.

## Results

**Fig. 1:**
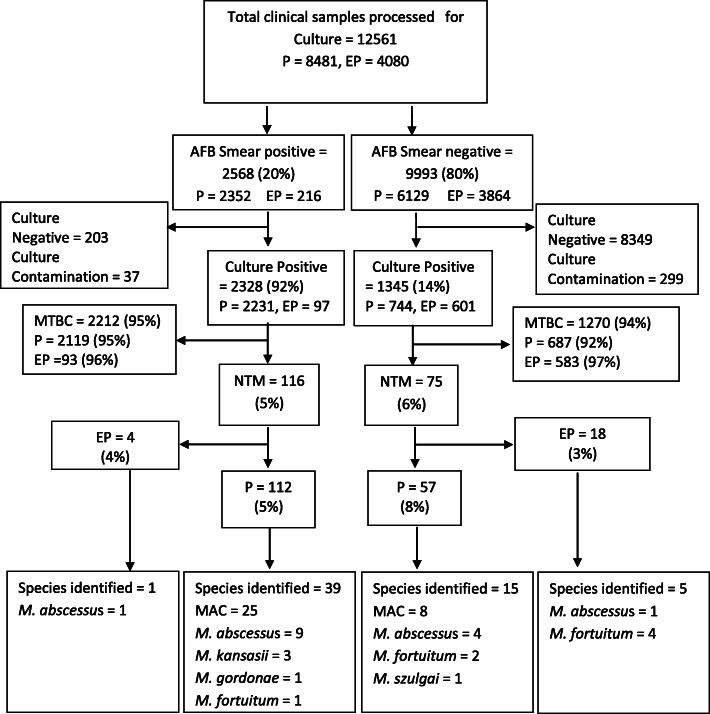
Flow diagram showing proportion of non-tuberculous mycobacteria isolated from AFB smear-positive and AFB-smear negative pulmonary and extrapulmonary specimens processed for culture from 2016 to 2019. AFB = acid fast bacilli, P = pulmonary, ExP = extrapulmonary, MTBC = *Mycobacterium tuberculosis* comple*x*, NTM = Non-tuberculous mycobacteria, MAC = *Mycobacterium avium* comple*x*

A total of 12,561 samples were received for culture during 2016–2019 (Fig. 1). Among these 2568 (20%) samples were positive for AFB on smear examination while 9993 (80%) were negative for AFB on smear. Among the smear-positive samples, 2328 (92%) were culture positive, 2212 (95%) of these were identified as MTBC, while 116 (5%) were declared NTM. Among the smear-negative samples, 1345 (14%) were culture positive, 1270 (94%) were MTBC and 75 (6%) were NTM.

### Species identification

Species identification was done for 60/191 samples (54 pulmonary and 6 extrapulmonary) as shown in Fig. 1. Among pulmonary samples, *M. avium* complex was identified in the majority 33/54 (61%), followed by *M. abscessus* 13/53 (24%), *M. fortuitum* 3/54 (5.5%), *M. kansasii* 3/54 (5.5%), *M. gordonae* 1/54 (2%) and *M. szulgai* 1/54 (2%). In 6 extrapulmonary samples, *M. abscessus *was isolated in 2 (33%) while *M. fortuitum* was isolated in 4 (67%) samples. Table 1 shows year wise data of the total cultures applied, and MTBC, and NTM isolated from the AFB positive isolates. NTM Species identification was done for 1/43 (2%), 12/70 (17%), 28/50 (56%), and 19/28 (68%) of NTM isolates reported respectively in 2016–2019.

**Table 1:** Specimens processed and prevalence of non-tuberculous mycobacteria among patients investigated for tuberculosis

Year	2016	2017	2018	2019	Total
Culture processed	1934	4781	3173	2673	12,561
Culture Positive for AFB	1165	875	944	689	3673
MPT64-positive (MTBC) n (%)	1122 (96)	805 (92)	894 (95)	661 (96)	3482 (95)
MPT64-negative (NTM) n (%)	43 (4)	70 (8)	50 (5)	28 (4)	191 (5)
NTM Speciation performed n (%)	1 (2)	12 (17)	28 (56)	19 (68)	60 (31)

*AFB* Acid fact bacilli, *MTBC Mycobacterium tuberculosis* complex, *NTM* Non-tuberculous mycobacteria, *n* number

### Patient characteristics

Table 2 shows the characteristics of patients with culture-positive pulmonary (*n* = 169) and extrapulmonary disease (*n* = 22) caused by NTM. Human immunodeficiency virus (HIV) status was available for 22 cases and all were negative.

**Table 2:** Characteristics of patients with positive culture for non-tuberculous mycobacteria

	Pulmonary	Extrapulmonary	*p*-value
Total Patients, n/N (%)	169/191 (88)	22/191 (12)	
Median Age, years (Range)	45^d^ (6–75)	21 (6–90)	< 0 .001
**Gender,** n/N (%)			
Male	103/169 (61)	7/22 (32)	0.009
Female	66/169 (39)	15/22 (68)	
**Treatment history**^**a**^**,** n/N (%)			
Single cycle of RHEZ^b^	82/169 (48)	2/22 (9)	< 0 .001
One or more cycles of RHEZS^c^	28/169 (17)	0/22 (0)	
Unknown	34/169 (20)	5/22 (23)	
Not Treated	25/169 (15)	15/22 (68)	
**HIV Status,** n/N (%)			
Negative	22/169 (13)	0/22 (0)	0.936
Unknown	147/169 (87)	22/22 (100)	
**Sites involved,** n/N (%)			
Lung	169/169 (100)	–	
Lymph nodes	–	7/22 (32)	
Pleural fluid	–	8/22 (36)	
Pus	–	3/22 (14)	
Tissue	–	3/22 (14)	
Perinephric fluid	–	1/22 (4)	

*n* number, *N* total number, *%* percentage, *MAC Mycobacterium avium* complex

^a^Previous anti-tuberculous treatment

^b^RHEZ = rifampicin + isoniazid + ethambutol + pyarzinamide

^c^RHEZPS = rifampicin + isoniazid + ethambutol + pyrazinamide + streptomycin

^d^Data from163 patients, missing data for age for 3 patients

Among the pulmonary NTM disease, there was a male preponderance (61%), whereas extrapulmonary NTM disease was more common in females (68%). Among the pulmonary NTM disease, 110/169 (65%) had received anti-TB treatment before their specimens were tested for culture. In these cases, culture was requested by the physicians to rule out drug resistance TB. Among these 82/110 (75%) had received 2 months of treatment with isoniazid (H), rifampicin (R), ethambutol (E), and pyrazinamide (Z) and 4 months with RHE. Streptomycin was given in addition to 28/110 (25%) patients during the first 2 months. In extrapulmonary NTM disease, the majority (15/22, 68%) were never treated for TB. In these cases, culture was requested as a part of diagnostic work-up. Among 22 NTM reported in the extrapulmonary specimens, the disease sites included lymph nodes in 7, pleural fluids in 8, and perinephric abscess in 1. Disease sites were not specified in 6 samples, labeled as pus (3) and other tissue samples (3).

### Case-based data

Case-based data with complete medical records and follow-up details were available from only12 patients as shown in Table 3.

**Table 3:** Clinical features, drug susceptibility and treatment outcome for a subgroup of 12 female patients with non-tuberculous mycobacterial disease

Pat/N	Age (years)	Site	symptoms	CXR- Finding	Co-morbidities	NTM-specie	DST Profile	TM Regimen	Follow-up AFB smears and cultures					TM duration	TM outcome
									2 M	4 M	6 M	9 M	End of TM		
1	20	Pul	F, WL, PC, CP	BL/FCD	None	MAC	CLR, MXF, AMK	EHR, CLR, AMK (6 M)	P/P	P/P	N/NG	N/NG	N/NG	18 M	Cured
2	50	Pul	F, WL, PC, HM	BL/FCD	CLD, DM	MAC	CLR, MXF, AMK	EHR, CLR, AMK (6 M)	P/P	P/P	N/NG	N/NG	N/NG	18 M	Cured
3	64	Pul	F, WL, PC	BL/FCD	DM	MAC	CLR, MXF, AMK,LZD	EHR, CLR AMK (6 M)	P/P	P/P	N/NG	N/NG	N/NG	18 M	Cured
4	48	Pul	F, HM, SOB	LT/FCD	None	MAC	CLR, MXF ***AMK(r)***^a^	EHR, CLR,	P/P	NA	NA	N/NG	N/NG	18 M	Cured
5	45	Pul	F, WL, PC	BL/FCD	CLD, DM	MAC	CLR, MXF, AMK	EHR, CLR, AMK (6 M)	P/P	P/P	N/NG	N/NG	N/NG	18 M	Cured
6	45	Pul	F, WL, PC	BL/FCD	CLD	MAC	CLR, MXF, AMK	EHR, CLR, AMK (6 M)	P/P	P/P	N/NG	N/NG	N/NG	18 M	Cured
7	54	Pul	F, WL, PC, HM	BL/FCD	DM	MAC	CLR, MXF, AMK	EHR, CLR, AMK (6 M)	P/P	P/P	N/NG	N/NG	N/NG	18 M	Cured
8	65	Pul	F, WL, PC	BL/FCD	DM	MAC	CLR, MXF, AMK	EHR, CLR, AMK (6 M)	P/P	P/P	N/NG	N/NG	N/NG	18 M	Cured
9	68	Pul/ LN	F, WL, PC	BL/FCD	HCV+	MAC	CLR, MXF, AMK	EHR, CLR, AMK (6 M)	P/P	N/NG	N/NG	N/NG	N/NG	18 M	Cured
10	18	Pul	F, WL, PC	BL/FCD LT/PTX	None	*M. abscessus*	MXF, LZD, CLR	EHR+ CLR (6 M)-shifted to MXF+ LZD (3 M)	P/P	P/P	P/P	Expired	–	8 M	Died
11	65	Pul	F, PC	BL/FCD	None	*M. abscessus*	MXF, LZD, CLR	LZD, MXF AMK (3 M)	P/P	P/P	P/P	N/P	NA	12 M	LTFUP
12	32	Pul	F, WL, PC	LT/FCD	None	*M. abscessus*	MXF, LZD, CLR	MXF, LZD CLR (2 M)	P/P	P/P	N/NG	N/NG	N/NG	18 M	Cured

*Pat* patient, *N* number, *CXR* chest X-ray, *NTM* Non-tuberculous mycobacteria, *DST* Drug susceptibility testing, *TM* treatment, *M* month, *Pul* pulmonary, *LN* Lymph node, *F* Fever, *WL* Weight loss, *PC* Productive cough, *CP* Chest pain, *SOB* Shortness of breath, *HM* Hemoptysis, *BL* Bilateral, *FCD* Fibro-cavitary disease, *LT* Left, *PTX* Pneumothorax, *CLD* Chronic liver disease, *DM* Diabetes mellitus, *HCV* Hepatitis C virus, *MAC Mycobacterium avium* complex, *CLR* Clarithromycin, *MXF* Moxifloxacin, *AMK* Amikacin, *LZD* Linezolid, *EHR* Ethambutol + isoniazid + rifampicin, *r* Resistant, ^a^resistant to amikacin (patient-4). *P* Positive, *N* Negative, *NG* No growth, *NA* Not available, *LTFUP* Lost to follow-up

#### Symptoms and radiology

All 12 patients for whom complete data were available were females suffering from pulmonary disease, with concomitant involvement of lymph nodes in one patient. All patients presented with fever, productive cough, and weight loss. Hemoptysis was present in 3/12 patients, but none had any life-threatening episodes. All had extensive fibro-cavitary disease on radiology and long-term oxygen therapy was required by only one patient.

#### Comorbid conditions

Among these patients, concomitant diabetes was present in 5/12 (42%), chronic liver disease in 3/12 (25%). Two (17%) patients had both diabetes and chronic liver disease.

#### Laboratory investigations

Sputum specimens from all 12 patients were positive for AFB smear and MTB was not detected on Xpert MTB/RIF assay. Mycobacterial cultures from these patients’ specimens yielded AFB positive isolates, negative for MTBC. Nine of these patients had *M.avium* complex and 3 had *M. abscessus* disease.

#### Phenotypic drug susceptibility testing

All nine *M. avium* complex isolates were tested for clarithromycin, amikacin, and moxifloxacin, and among these only one case was resistant to amikacin. All three *M. abscessus* isolates were sensitive to clarithromycin, moxifloxacin, and linezolid.

#### Treatment

All cases of *M. avium* complex were treated with ethambutol, rifampicin, isoniazid (RHE combination as per weight), clarithromycin (500 mg BD), and amikacin (500 mg BD) IM thrice weekly for initial 6 months (except for one case resistant to amikacin, who was treated without it). Treatment was given for 18 months. Isoniazid was added in therapy as rifampicin and ethambutol were only available in combination with isoniazid. All cases of *M. abscessus* were treated with moxifloxacin and linezolid. Among these, patient 10 received RHE and clarithromycin initially till results of species identification and drug susceptibility testing became available, patient 11 stopped amikacin after 3 months due to side effects, and patient 12 stopped clarithromycin after 2 months due to resistance.

#### Follow-ups

All 12 patients were monitored for treatment response and adverse drug reactions. Patients were called weekly for the first 2 months and then monthly for the rest of the treatment period for complete blood picture, renal and liver function tests. Visual acuity and ECGs were checked on the first follow-up only. These patients were followed-up by the doctor at 2, 4, 6, and 9 months and if culture-negative, the last culture was performed at the end of the treatment.

#### Treatment response

No serious side effects were observed during the treatment. All patients started improving symptomatically after 2 months of treatment. One patient was culture-negative at 4 months while nine more became culture-negative at 6 months. Two patients remained culture-positive after 6 months of treatment. Among those two who were culture-positive at 6 months, one remained culture-positive at 9 months and did not come for further follow-ups after 12 months of treatment. The second one, who was culture-positive at 6 months, expired during the eighth month of treatment, This patient developed respiratory failure type 1 and required long-term oxygen therapy, developed left-sided pneumothorax which resolved with treatment, but respiratory failure worsened with an increase in requirement for oxygen therapy. The patient expired at home after 1 month of discharge. Ten patients who completed treatment remained culture-negative after conversion and were declared cured at the end of the treatment.

Generally, the disease was more severe among patients infected with *M. abscessus.* Symptoms in these patients took longer to resolve and culture conversion was late, where 2/3 of these patients remained culture-positive at 6 months. The treatment outcome was also less favorable in these patients.

## Discussion

Our study shows that 5% of the culture-positive patients who were initially presumed to have TB or were treated for TB, were infected with NTM. More than 65 % of these patients had received anti-TB treatment, and a small but significant proportion had received multiple cycles of anti-TB therapy. These findings show that a substantial number of patients with NTM disease in the high TB prevalence settings with large populations are incorrectly diagnosed as TB, leading to inappropriate treatment, unfavorable outcomes, and increased morbidity. In settings with limited access to culture or nucleic acid amplification testing, the risk is further augmented when patients fail to respond to standard anti-TB therapy with a likelihood of prompting empirical treatment recommended for drug-resistant TB. Empirical treatment with second-line anti-TB drugs in such scenarios would cause further delays in the correct diagnosis and increased morbidity due to adverse drug effects. This highlights the importance of confirmation of AFB seen on microscopy as MTBC with nucleic acid amplification testing, and further investigations including culture with species identification for all cases reported positive for AFB microscopy but are negative for MTBC. Diagnosis of NTM infection requires repeated cultures as in the international guidelines for NTM lung disease [18], highlighting the need to strengthen laboratory facilities for mycobacterial cultures. MPT64 based tests are shown to be a rapid and reliable method for MTBC identification in liquid culture assays [19]. A small minority of MTBC isolates are not detected by MPT64 assays due to deletion or mutation of the MPT64 gene (absent from some *M. bovis* BCG strains) [20–22], or due to low MPT64 concentrations in early cultures or mixed cultures [23, 24]. The low cost and simplicity of MPT64 assays would outweigh the small reduction in sensitivity in most settings and performing nucleic acid amplification tests on MPT64-negative isolates might further improve sensitivity [22].

*M. avium* complex is reported to be the predominant species in the pulmonary NTM cases, constituting up to 80% of NTM infections [25], however, in our population, *M. avium* complex was isolated in 60% of pulmonary cases, whereas *M. abscessus* was reported in 24% of cases. This difference in prevalent NTM causing pulmonary disease could have been due to our small sample size. It has been reported previously that *M.abscessus* is shown to be more prevalent among patients with cystic fibrosis [26]. None of the patients in our cohort was known to have cystic fibrosis implying that *M. abscessus* should be considered a pathogen among non-cystic fibrosis patients as well. Disease caused by *M. abscessus* is more aggressive leading to irreversible loss of lung function [27], and timely and accurate diagnosis and treatment could lead to preservation of lung function.

Studies have shown a female preponderance for *M. avium* pulmonary infections. Older females of thin body type with otherwise healthy lungs are shown to have *M. avium* complex infection mainly in the middle portions of their lungs. In contrast, we report a male preponderance (61%) in our study population. Smoking is a known risk factor for chronic obstructive lung disease (COPD), and COPD could predispose to NTM infections and it increases the decline in lung function in COPD patients [28–30]. Smoking is much more prevalent among males as compared to females in Pakistan and this comorbidity among males could be a possible explanation for observed male predominance, but we do not have enough data to test this hypothesis.

Even though NTM disease is said to occur more frequently in the HIV-positive population, there were no HIV-positive patients among the 20% tested for HIV in our cohort. Considering the low prevalence of HIV in Pakistan [31], the findings from this study may be considered applicable to HIV-negative populations.

We studied clinical records available for a subset of 12 patients. Drug susceptibility results were available for all 12 patients and the susceptibility profile reported was similar for nine patients with NTM disease caused by *M. avium* complex and three with *M.abscessus*. Uniform drug susceptibility profile for a particular NTM species suggests that patients can be treated based on speciation results of NTM. We also studied the clinical records of these 12 patients for treatment regimen, clinical and bacteriological response, and treatment outcomes. Treatment success was high for patients with *M. avium* complex despite fibro-cavitary disease as compared to other studies [32, 33]. All patients reported good clinical response with resolution of symptoms by the end of the second month of treatment and were culture negative by the end of the sixth month of treatment. One reason could have been the continuation of quadruple therapy and the uninterrupted use of intramuscular amikacin for the initial 6 months. However, due to the small sample size, a firm conclusion cannot be made. Compared to *M. avium* complex, patients with *M. abscessus* although younger, showed a relatively poor clinical response and unfavorable outcomes, and culture conversion took a longer time as shown in the previous studies [34].

Prevalence of diabetes (5/12, 42%) was higher in our patient group compared to the general population [35] showing that patients with diabetes may be more susceptible to NTM infections. Our findings are consistent with other studies showing increased susceptibility to non-respiratory NTM infections in patients with diabetes mellitus [36, 37]. Diabetes is a multifactorial metabolic condition with a complex etiopathology. Exposure to chronic hyperglycemic conditions can induce oxidative stress, inflammatory changes, and immune dysfunction, contributing to increased susceptibility to bacterial infections along with a poorer prognosis [38–40]. The prevalence of diabetes is much higher in the resource-poor regions where TB and environmental NTM infections are both highly endemic and where diagnostic facilities are rudimentary [41–43]. The increased susceptibility to bacterial pathogens in diabetes represents an important complication given the growing combined burden of communicable and non-communicable conditions [38, 44].

Our study has some limitations. First, this study was conducted in a specialized tertiary care hospital, and the possibility of a higher prevalence of NTM compared to the general population cannot be excluded because of a higher proportion of patients with a history of previous TB treatment, poor response to TB treatment or difficulty in diagnosis, seeking care from this hospital. Secondly, the analysis was based on patient samples selected for culture, and this could have added the selection bias. Thirdly, because of the retrospective nature of the study, the information collected and clinical record retrieved were not complete, limiting the generalizability of the findings. Lastly, a comparison of treatment outcomes was not possible between the patients diagnosed promptly and those who faced diagnostic and treatment delays due to misdiagnosis. This limitation can be addressed in future prospective studies to compare the outcomes between these two groups and to see the impact of diagnostic delays in the initiation of treatment in NTM disease. Despite these limitations, the findings from this study add to the information on the prevalence of NTM disease in low HIV and high TB endemic settings and highlight the need to improve diagnosis and treatment of NTM disease.

## Conclusion

This study shows a 5% prevalence of NTM among presumptive TB patients investigated for tuberculosis in a tertiary care hospital. Considering the high prevalence of TB in Pakistan, the risk of patients being misdiagnosed and receiving inappropriate treatment can be substantial. Our study highlights the need to strengthen and expand clinical and laboratory services to diagnose and manage diseases caused by NTM in parallel with MTBC.

## Data Availability

The datasets generated during and/or analyzed during the current study are available from the corresponding author on reasonable request.

## Abbreviations

NTM: Non-tuberculous mycobacteria
AFB: Acid-fast bacilli
TB: Tuberculosis
MTBC: *Mycobacterium tuberculosis* complex
HIV: Human immunodeficiency virus
COPD: Chronic obstructive lung disease
